# Ar–Ar dating for hydrothermal quartz from the 2.4 Ga Ongeluk Formation, South Africa: implications for seafloor hydrothermal circulation

**DOI:** 10.1098/rsos.180260

**Published:** 2018-09-26

**Authors:** Takuya Saito, Hua-Ning Qiu, Takazo Shibuya, Yi-Bing Li, Kouki Kitajima, Shinji Yamamoto, Hisahiro Ueda, Tsuyoshi Komiya, Shigenori Maruyama

**Affiliations:** 1 Department of Subsurface Geobiological Analysis and Research (D-SUGAR), Japan Agency for Marine-Earth Science and Technology (JAMSTEC), Kanagawa 237-0061, Japan; 2 Research and Development Center for Submarine Resources, Japan Agency for Marine-Earth Science and Technology (JAMSTEC), Kanagawa 237-0061, Japan; 3 Project Team for Development of New-Generation Research Protocol for Submarine Resources, Japan Agency for Marine-Earth Science and Technology (JAMSTEC), Kanagawa 237-0061, Japan; 4 Key Laboratory of Tectonics and Petroleum Resources (China University of Geosciences Wuhan), Ministry of Education, Wuhan 430074, People's Republic of China; 5 Institute of Geology, Chinese Academy of Geological Sciences, Beijing 100037, People's Republic of China; 6 Department of Geoscience, University of Wisconsin-Madison, Madison, WI 53706, USA; 7 Graduate School of Environment and Information Sciences, Yokohama National University, Yokohama 240-8501, Japan; 8 Department of Earth and Planetary Sciences, Tokyo Institute of Technology, Tokyo 152-8551, Japan; 9 Department of Earth Science and Astronomy, The University of Tokyo, Tokyo 153-8902, Japan; 10 Earth-Life Science Institute, Tokyo Institute of Technology, Tokyo 152-8550, Japan

**Keywords:** fluid inclusion, Ar–Ar dating, hydrothermal quartz, Ongeluk Formation, hydrothermal alteration, South Africa

## Abstract

Fluid inclusions in hydrothermal quartz in the 2.4 Ga Ongeluk Formation, South Africa, are expected to partially retain a component of the ancient seawater. To constrain the origin of the fluid and the quartz precipitation age, we conducted Ar–Ar dating for the quartz via a stepwise crushing method. The obtained argon isotopes show two or three endmembers with one or two binary mixing lines as the crushing proceeds, suggesting that the isotopic compositions of these endmembers correspond to fluid inclusions of each generation, earlier generated smaller ^40^Ar- and K-rich inclusions, moderate ^40^Ar- and ^38^Ar_Cl_ (neutron-induced ^38^Ar from Cl)-rich inclusions and later generated larger atmospheric-rich inclusions. The K-rich inclusions show significantly different ^40^Ar/^38^Ar_Cl_ values compared to the ^38^Ar_Cl_-rich inclusions, indicating that it is difficult to constrain the quartz formation age using only fluid inclusions containing excess ^40^Ar. The highest obtained ^40^Ar/^36^Ar value from the fluid inclusions is consistent with an expected value of the Ongeluk plume source, suggesting that the quartz precipitation was driven by Ongeluk volcanism. Considering the fluid inclusion generations and their compositions, the hydrothermal system was composed of crustal fluid and magmatic fluid without seawater before the beginning of a small amount of seawater input to the hydrothermal system.

## 1. Introduction

It is believed that the evolution of life has been frequently influenced by changes in the surface environment throughout Earth's history (e.g. [1–3]). As revealed by fossil records, several destructive environmental changes have induced mass extinctions and triggered increases in the diversity of life [4,5]. In particular, global glaciation (Snowball Earth), which has occurred a few times in Earth's history [6,7] could probably apply intense selective pressure on life to evolve [8]. In addition to extreme cooling, the seawater compositions were probably drastically changed by the formation of voluminous ice sheets on land and the isolation between the atmosphere and the oceans, which would also behave as a selective pressure. Therefore, to consider the factors contributing to the evolution of life before and after Snowball Earth events, the compositional changes of seawater need to be estimated from geological records.

One of the best methods to estimate the compositions of ancient seawater is the study of fluid inclusions in hydrothermal quartz precipitated in drainage cavities and interstitial spaces between seafloor pillow lavas because such hydrothermal quartz is presumably formed via mixing between the subseafloor hydrothermal fluid and seawater [9–17]. However, the timing of this event, i.e. whether the hydrothermal quartz precipitated at the time of seafloor hydrothermal circulation or during later thermal events, has frequently been debated (e.g. [18]). Furthermore, the origin of the trapped fluid as a fluid inclusion needs to be constrained to estimate the palaeo-seawater composition because the trapped fluid potentially has various origins: not only seawater and magmatic fluid, but also meteoric water and crustal fluid.

Previously, to provide constraints on the formation ages of quartz-bearing hydrothermal ore deposits, Ar–Ar dating for fluid inclusions and trapped minerals within quartz has been conducted via crushing and heating methods (e.g. [19–23]). In general, ^40^Ar has many reservoirs, such as the atmosphere and metamorphic fluid. ^40^Ar, except atmospheric ^40^Ar (^40^Ar_A_) and radiogenic ^40^Ar (^40^Ar_R_), is referred as excess ^40^Ar (^40^Ar_E_). The ubiquitous ^40^Ar_E_ in natural samples often causes considerable difficulties in Ar–Ar dating. To overcome this problem, several studies have provided useful methods for data analysis, e.g. isochron diagrams for stepwise crushing analyses of fluid inclusions (e.g. [21,23]), and three-dimensional (3D) multi-component correlation diagrams for the stepwise crushing of fluid inclusions and the heating of impurity minerals (e.g. [19,20,22]). The calculations of these methods simultaneously provide the ratio between the sum of ^40^Ar_A_ and ^40^Ar_E_ (^40^Ar_A+E_) and the atmospheric ^36^Ar (^36^Ar_A_). The ^40^Ar_A+E_/^36^Ar_A_ value can be used to constrain the fluid origins because their sources have unique values, e.g. continental crust (70 000; [24–26]), mid-ocean ridge basalt (28 000; [27]; 40 000; [28]) and oceanic island basalt (approximately 8000; [29]). Recently, attempts have been made to estimate Archaean atmospheric ^40^Ar/^36^Ar value and formation age by Ar–Ar dating using fluid inclusions trapped in hydrothermal quartz extracted by the crushing and heating methods [30,31]. These estimations are based on an assumption of constant ^40^Ar_E_/^38^Ar_Cl_ value in all fluid inclusion. However, it is unclear whether all fluid inclusions contain a similar ^40^Ar_E_/^38^Ar_Cl_ value in the single sample.

In this study, we focused on the 2.4 Ga Ongeluk Formation, Transvaal Supergroup, South Africa. This formation is composed of submarine lava flows that are thought to have erupted during the Palaeoproterozoic Snowball Earth Event, as suggested by palaeomagnetic studies and geological field observations [7,32–34]. The lavas preserve the ancient seafloor hydrothermal alteration [33,35,36] and the hydrothermally precipitated quartz in the Ongeluk Formation is strongly suggested to have formed at the time of seafloor hydrothermal circulation [12,13,35]. Furthermore, the chemical compositions of the fluid inclusions in the quartz samples indicate that the fluid inclusions partially contain the seawater component at the time of seafloor hydrothermal circulation [12,13,35]. However, it is important to assess the likelihood that the seawater component is truly derived from the 2.4 Ga Ongeluk seawater based on the formation age of the hydrothermal quartz and to constrain the sources of the other fluid components. Therefore, we carried out Ar–Ar dating with stepwise crushing techniques to determine the formation age and to constrain the source(s) of the inclusion fluid in the quartz samples collected from the Ongeluk Formation.

## 2. Geological setting

**Figure 1.**
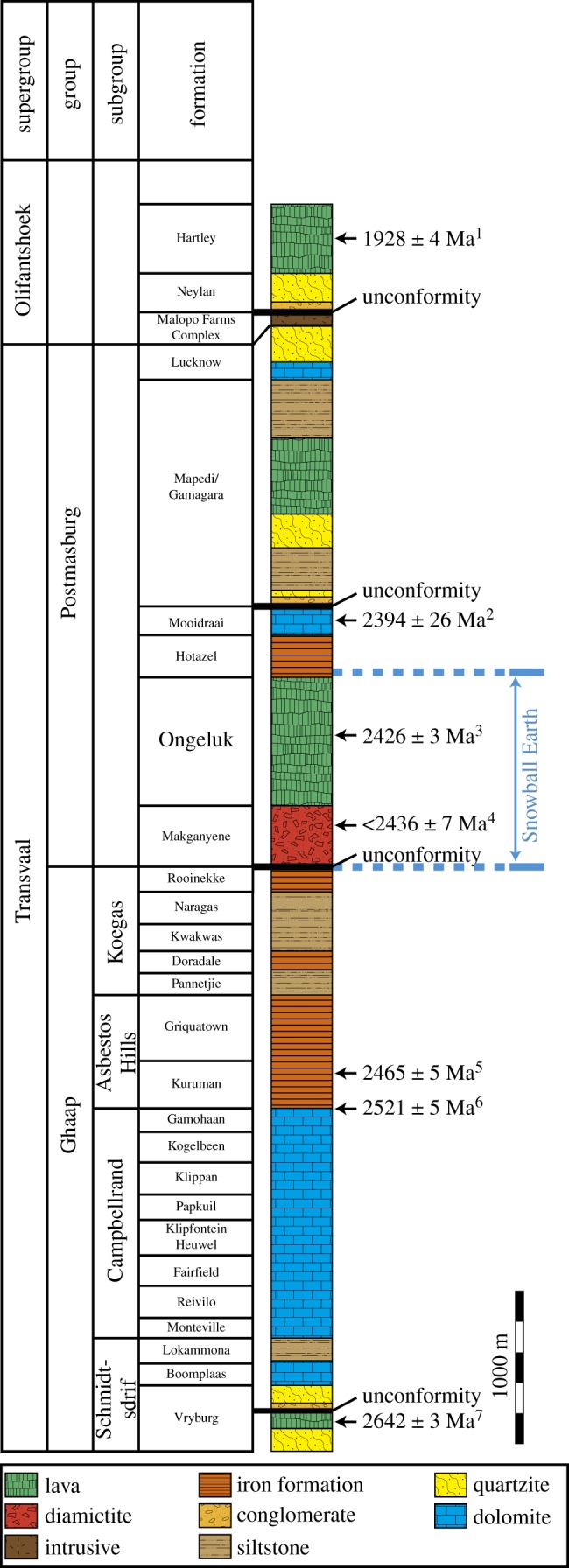
Simplified stratigraphic column section of the Transvaal and Olifantshoek Supergroups in the Griqualand West Basin, South Africa (modified after [38]). ^1^Zircon Pb–Pb age [39]. ^2^Carbonate U–Pb and Pb–Pb age [40].^3^ID–TIMS U–PB baddeleyite age [41]. ^4^Detrital zircon U–Pb youngest age [42]. ^5^SHRIMP zircon U–Pb age [43]. ^6^Zircon U–Pb age [44]. ^7^Single zircon Pb-evaporation age [45].

**Figure 2.**
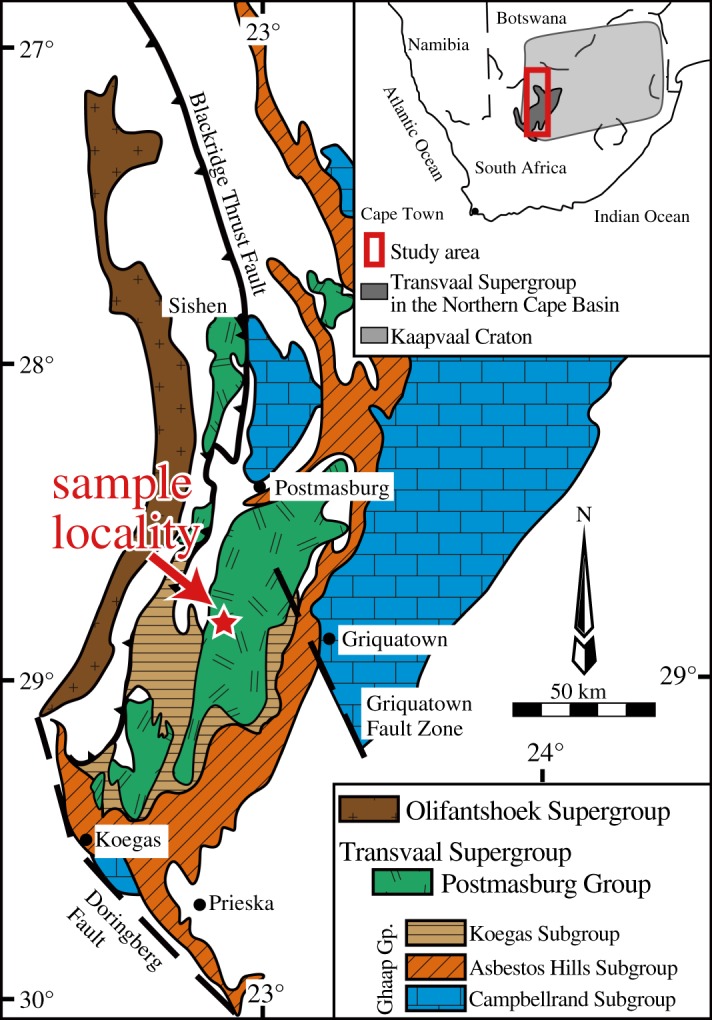
Geological map of the Griqualand West Basin in the Northern Cape Province showing the distribution of the Postmasburg Group (including the Ongeluk Formation). A star indicates the sampling location: the Bosh Aar farm. The map modified after [46].

**Figure 3.**
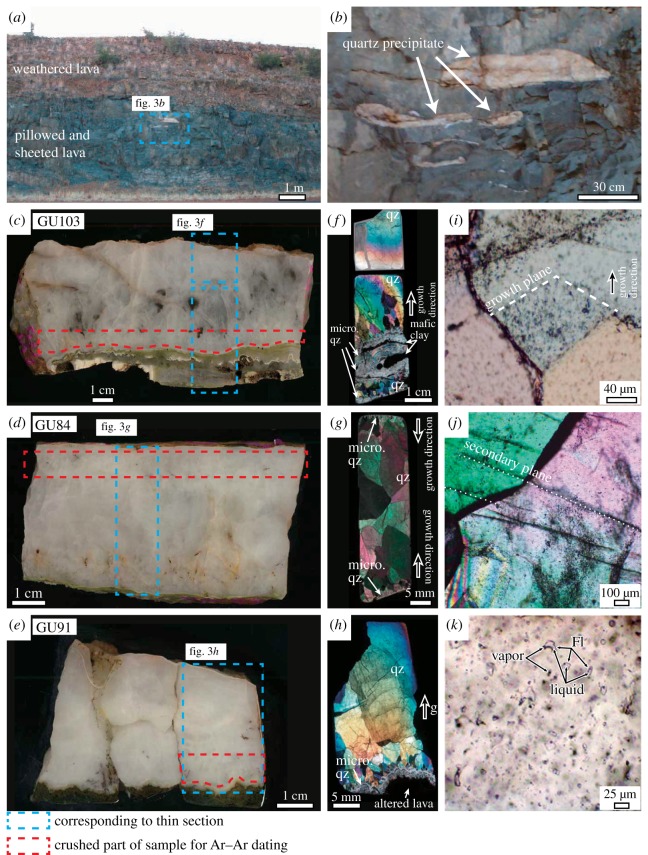
(*a*) Photograph of the sheeted/pillowed basalts that out crop at the sampling locality. The dark green coloured basalts preserve the igneous structures, whereas the upper brownish basalt is highly weathered. (*b*) Drainage cavities filled with hydrothermal quartz (the white parts) correspond to the area in the blue broken line in panel (*a*). (*c–e*) Photographs of the quartz slabs for samples GU103, GU84 and GU91, respectively. The white parts consist of crystallized quartz, while the brown parts contain not only quartz but also oxidized pyrite. The red broken lines in each panel indicate the positions of the samples used for the argon analysis. (*f–h*) Photomicrographs (crossed Nicol) of thin sections corresponding to each area in the blue broken lines in (*c–e*), respectively. The light grey area in the lower part of (*f*) is composed of fine-grained quartz (micro. qz) intercalated with clay minerals corresponding to the light green area in the lower area of (*c*). The grain sizes of the quartz in (*f*,*h*) become larger from the bottom to the top, indicating upward growth in these thin sections. The grain sizes of the quartz close to the rims in panel (*g*) are smaller than those in the centre, indicating that the growth direction is from the rim to the centre. Secondary cracks are minor in (*f*,*g*); however, a relatively large amount of secondary cracks are observed in (*h*). (*i*) Photomicrograph of a quartz sample with crossed Nicol showing the primary fluid inclusions as black dots distributed along the growth planes of a quartz crystal. (*j*) Photomicrograph of a quartz sample with crossed Nicol showing the secondary fluid inclusions as black dots along secondary healed cracks crossing over a grain boundary between two quartz crystals. (*k*) Photomicrograph of a quartz sample with open Nicol showing the liquid-dominated primary fluid inclusions (FI) distributed randomly.

The 2.4 Ga Ongeluk Formation belongs to the Postmasburg Group, Transvaal Supergroup in the Griqualand West Basin, Kaapvaal Craton, South Africa. The Kaapvaal Craton extends over an area of approximately 1.2 million square kilometres, chronologically spanning from 3.6 to 1.9 Ga. The lower part is composed of granite–greenstone belts that were unconformably covered by the Dominion Group, Witwatersrand Supergroup, Ventersdorp Supergroup, Transvaal Supergroup and Olifantshoek Supergroup in ascending stratigraphic order. The Transvaal Supergroup is a platform succession that covered the Kaapvaal Craton from the Late Archaean to the Palaeoproterozoic [37] (figure 1). The Transvaal Supergroup was deposited in three different basins: the Griqualand West Basin, Transvaal Basin and Kanye Basin. The Griqualand West Basin located in the Northern Cape Province is usually subdivided into the Ghaap Group and the overlying Postmasburg Group (figures 1 and 2). The Ghaap Group primarily consists of clastic rocks, carbonates, chert and banded iron formations, chronologically spanning from 2642 ± 3 to 2465 ± 5 Ma [43–47]. The Postmasburg Group consists of the Makganyene Formation, Ongeluk Formation, Hotazel Formation and Mooidraai Formation in ascending stratigraphic order [47]. The Makganyene Formation consists of a glacial diamictite and underlies the basaltic andesite lavas of the Ongeluk Formation (figure 3*a*). The clastic rocks in the Makganyene Formation do not contain detrital zircons younger than 2.4 Ga [42]. The age of the dolerites and basalts in the Ongeluk Formation was estimated to be 2425.5 ± 2.6 Ma from U–Pb dating of baddeleyite within the formation [41]. The Hotazel Formation conformably covers the Ongeluk Formation and consists of banded iron and manganese formations. The Mooidraai Formation contains dolostone, shale, quartzite and lava. The dolostone of the Mooidraai Formation gives a secondary-lead Pb–Pb age of 2394 ± 26 Ma [40]. The Mapedi Formation unconformably overlies the Mooidraai Formation, which is composed of basal conglomerate, lavas and shales covered by arenites of the Lucknow Formation.

The Ongeluk Formation is a thick succession (up to 900 m) of pillowed/sheeted basaltic andesite and hyaloclastite (figure 3*a*), which erupted in a shallow marine environment along the submerged western margin of the Kaapvaal Craton [33,48]. The Ongeluk lava apparently conformably overlies the Makganyene Formation (diamictite) with no palaeo-weathering [32,49]. The palaeomagnetic data for the Ongeluk lavas show a depositional palaeolatitude of 11 ± 6°, indicating that the Makganyene was deposited in tropical latitudes [32,41]. The presence of dropstones of likely glacial origin at the base of the Hotazel Formation overlying the Ongeluk lavas indicates that the glaciation even lasted to after the Ongeluk volcanism [7,34,50] (figure 1).

The Ongeluk lavas have been hydrothermally altered and metamorphosed; the occurrence of pumpellyite in the Ongeluk lavas indicates that the prehnite-pumpellyite facies are probably the peak metamorphic condition [12,51,52]. Geochemical studies of the Ongeluk volcanic rocks have revealed the preservation of subseafloor hydrothermal alterations [33,35,36]. The hydrothermal alteration of the basaltic andesites preserves two types of alteration: high-temperature alteration causing K-depletion in the cores of the pillowed lavas and low-temperature alteration leading to K enrichment in the hyaloclastites and rims of the pillows [33].

## 3. Petrographic descriptions

**Figure 4.**
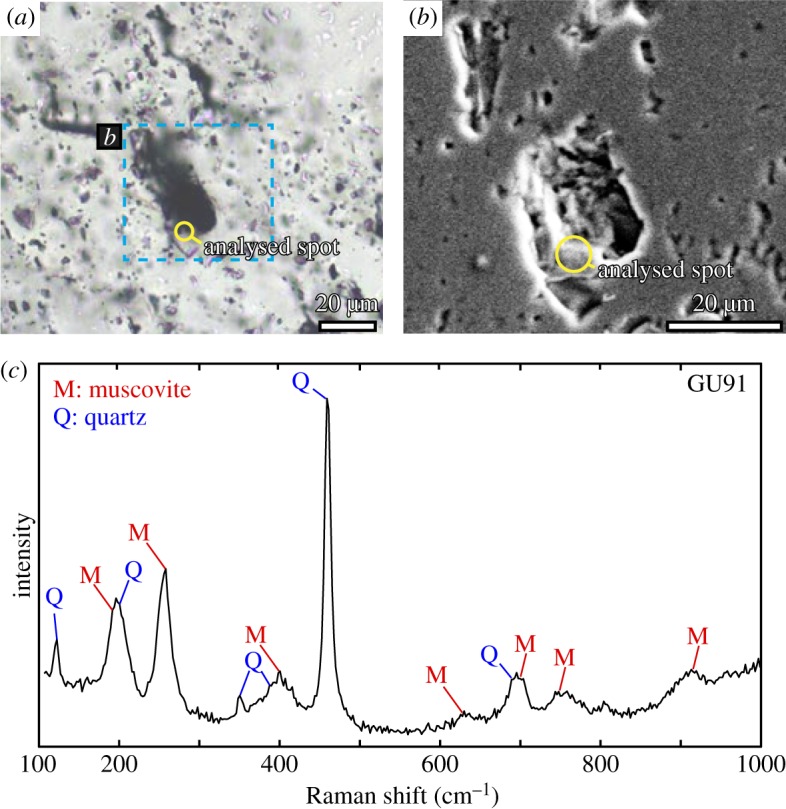
(*a*) Photomicrographs of a muscovite inclusion in quartz with transmitted light. The analysed spot is shown as an open yellow circle. The area surrounded by a blue broken line corresponds to (*b*). (*b*) Secondary electron microimage of a muscovite inclusion in quartz. (*c*) Representative Raman spectra of a muscovite inclusion within a quartz crystal. The major peaks of the obtained spectra are well fitted by a mixture of quartz and muscovite. The inclusion crystal was also qualitatively confirmed to be K–Al–silicate via the electron microprobe analysis.

The hydrothermal quartz samples analysed in this study were collected from the outcrops of the Ongeluk pillowed/sheeted lavas exposed along a road cut in the Bosch Aar farm (figure 2). As previously described in detail [12,13,35], the Ongeluk pillow lavas have original open spaces such as drainage and interpillow cavities, whose sizes range from approximately 10 to 50 cm long. They are filled with quartz and/or jasper precipitates [12,13,35] (figure 3*a,b*). The cavities are isolated from one another and never cut the pillow rims. The quartz precipitates consist primarily of milky quartz hosting multiple fluid inclusions, and the coarse-grained quartz crystals have needle-shaped pyrite with oxidized surfaces in some cases (the black part in figure 3*c–e*). Some quartz crystals show point symmetrical structures growing from the surface to the centre of the cavity and forming banded growths formed by changes in the grain size or the intercalation of fine-grained clay material [13] (figure 3*c–h*). The clay minerals probably originate from the chilled margins of the pillow lavas and the surface of drainage cavities, which are presumably rich in K as revealed by a previous study [33]. Indeed, fine-grained (less than 10 µm wide and a few µm thick) muscovite (sericite) has been identified in a 20 µm-sized fluid inclusion or in the quartz crystals via electron microprobe analysis and Raman spectroscopy (figure 4). These geological and mineralogical occurrences of quartz precipitates indicate their formation during low-temperature seafloor hydrothermal circulation [11,35]. Moreover, the compositional variation of the fluid inclusions in the quartz samples indicates that the seawater component is partially preserved in the fluid inclusions [12,13].

The hydrothermal quartz used in this study contains various sizes of fluid inclusions (from less than 1 to 40 µm, on average less than 10 µm). The fluid inclusions generally consist of liquid and vapour phases without a liquid CO_2_ phase at room temperature, and their liquid to vapour ratios range from 5 to 12. No observations have indicated the presence of halite as a daughter mineral at room temperature [12,13,35]. These fluid inclusions have been categorized into primary and secondary inclusions based on detailed petrographic and microthermometric observations; the homogenization temperatures vary from 66.6 to 122.9°C for the primary fluid inclusion and from 86.1 to 160.0°C for the secondary inclusions, while the salinities vary from approximately 2500 to 4600 mmol kg^−1^ for the primary inclusions and from 1130 to 2100 mmol kg^−1^ for the secondary inclusions [13]. Similar trends have been observed in the previous study [12].

The three analysed samples (GU84, GU91 and GU103) were carefully selected for Ar–Ar dating from a few tens of quartz samples (figure 3*c–e*) according to the abundance of the primary fluid inclusions and the relative paucity of the secondary fluid inclusions. The crushed parts of the samples for Ar–Ar dating are composed primarily of fine-grained quartz crystals that precipitated in the earlier stage of the quartz mineralization without apparent discontinuity of crystal growth and contain smaller amounts of secondary inclusions (figure 3*c–e*).

## 4. Analytical method

The quartz samples close to basalt parts were sliced; these samples were the earliest generation and are secondary inclusion-poor based on wide-range sample observations (figure 3*c–e*). The sliced quartz samples were crushed to a grain size of less than 1 mm using a tungsten mill. The sieved grain fractions of a 30–60 mesh (0.50–0.25 mm) were ultrasonically cleaned using ultra-pure water and 2 N HNO_3_. The quartz grains without oxidized brown parts and basaltic clay, which are a possible source of K and often disturb Ar–Ar dating, were selected. The picked quartz grains were cleaned again in the same manner and dried on a cleaned bench. A total of 1.00 g of quartz grains for each sample was prepared for Ar–Ar dating. In addition, because GU103 contains both clear transparent quartz grains and smoky grains, this sample was separated into two subsamples: GU103-1 and GU103-2.

Our ^40^Ar/^39^Ar experiments were measured using a GV Instruments 5400^®^ mass spectrometer at the Guangzhou Institute of Geochemistry, Chinese Academy of Sciences. The mass spectrometer is equipped with a Faraday and an electron multiplier in ion counting mode. To monitor the irradiating condition and to evaluate irradiation parameter (the *J* value), the quartz samples (approx. 150 mg each) and flux monitor ZBH25 biotite in China with an age of approximately 132.7 Ma, were irradiated at the 49-2 reactor in Beijing for 48 h. The irradiated samples and a crushing pestle (218 g) were put in a crusher composed of a type 316L stainless steel tube with a hole that was 170 mm long and 28 mm in diameter, after cleaning the crusher thoroughly.

To decrease background signals, the vacuum lines for extraction and purification were baked for approximately 10 h at 150°C using a heating tape while the crusher was also baked for 10 h at 150°C with a furnace. The system blanks were measured on the electron multiplier at the start and end of each sample experiment and between every four steps. The blank signal levels were 0.003–0.009 mV for ^36^Ar, 0.0001–0.0005 mV for ^37^Ar, 0.0011–0.0022 mV for ^38^Ar, 0.0009–0.0020 mV for ^39^Ar and 1.5–3.2 mV for ^40^Ar. The Faraday sensitivity of this 5400Ar mass spectrometer is 1.19 × 10^−15^ mol mV^−1^. The sample/blank ratios that relate to the precision of the experiments and the detection limit ranged from 20 to 292 for ^39^Ar.

The pestle was repeatedly lifted and dropped to crush the samples via an external electric magnet that was controlled by an adjustable repeating-timer relay. The free-fall drop of the pestle was conducted from approximately 4 cm above the sample, and the number of pestle drops was counted automatically. The frequency of the single crushing adjusts at 2.5 Hz. The pestle drop frequency and the current level of the electromagnet were kept constant to generate a stable impact energy for a single drop. The number of the drops for each step was increased to obtain enough argon signals to measure because the mass of the gas extracted from a sample by a single drop decreases due to the decrease in the sample grain size as the experiment proceeds. The released gas was purified using two SAES NP10 Zr/Al getters at room temperature and at approximately 400°C, respectively. The purified gas was introduced to and analysed by mass spectrometer working with the Faraday for ^40^Ar of samples and the secondary electron multiplier (Balzers SEV217) for the others. The results were calculated via the software ArArCALC software (version 2.4) [53].

## 5. Results and discussion

### 5.1. Release patterns of the argon isotopes

**Figure 5.**
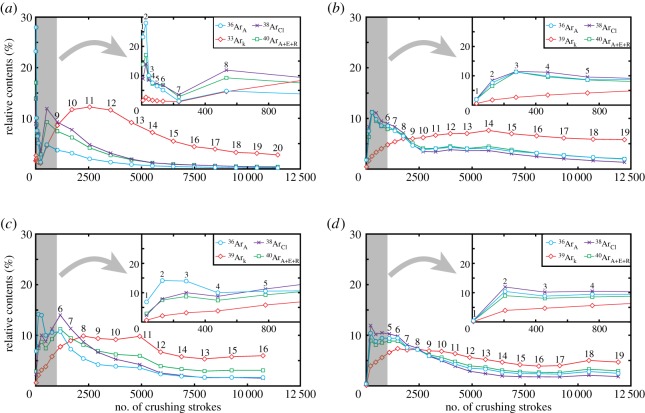
Degassing patterns of argon isotopes via *in vacuo* stepwise crushing of (*a*) GU84, (*b*) GU91, (*c*) GU103-1 and (*d*) GU103-2. The numbers above each point indicate the number of steps. The grey areas in each panel are enlarged on the upper right side. The degassing patterns for all samples show that most of the ^36^Ar_A_, ^38^Ar_Cl_ and ^40^Ar_A+E+R_ are released in the early steps while ^39^Ar_K_ is degassed primarily in the middle to later steps.

The release patterns of the argon isotopes provide information concerning the argon reservoirs in the quartz samples. For each sample, the ^39^Ar_K_ release pattern was clearly different from those of the other argon isotopes during the crushing extraction (table 1; figure 5). Taking GU84 as an example (figure 5*a*), the relative content of released ^36^Ar_A_ for the total amount of ^36^Ar_A_ extracted by approximately 12 000 crushing strokes was higher in the earliest steps (e.g. less than 100 strokes) than in the later steps while the relative contents of the released ^38^Ar_Cl_ and ^40^Ar_A+R+E_ broadly changed synchronously with that of ^36^Ar_A_. These results indicate that most of the ^36^Ar_A_, ^38^Ar_Cl_ and ^40^Ar_A+R+E_ were released in the early steps of the crushing experiment. Conversely, the relative content of ^39^Ar was generally low in the early steps, increased and reached the maximum value in the middle steps and then decreased thereafter. This release pattern of the four argon isotopes was also observed for other samples.

**Table 1.** Result of Ar–Ar dating analysis.

step	pestle drop numbers	total pestle drop numbers	^36^Ar_A_ ± 1*σ*	^38^Ar_Cl_ ± 1*σ*	^39^Ar_K_ ± 1*σ*	^40^Ar_A+R+E_ ± 1*σ*	^39^Ar_K_/^36^Ar_A_ ± 1*σ*	^40^Ar_A+R+E_/^36^Ar_A_ ± 1*σ*	^38^Ar_Cl_/^36^Ar_A_ ± 1*σ*	Ca/K ± 1*σ*
GU84										
1	8	8	0.005791 ± 0.000048	0.002259 ± 0.000016	0.000093 ± 0.000003	6.727295 ± 0.000246	0.016 ± 0.001	1161.7 ± 9.6	0.39 ± 0.00	6.11 ± 0.91
2	18	26	0.006977 ± 0.000058	0.003539 ± 0.000023	0.000155 ± 0.000003	8.273914 ± 0.000437	0.022 ± 0.000	1185.9 ± 9.8	0.51 ± 0.01	4.74 ± 0.63
3	18	44	0.002511 ± 0.000021	0.002156 ± 0.000011	0.000116 ± 0.000002	4.265796 ± 0.000235	0.046 ± 0.001	1699.1 ± 14.2	0.86 ± 0.01	3.51 ± 0.89
4	24	68	0.001889 ± 0.000017	0.001900 ± 0.000010	0.000096 ± 0.000003	3.532614 ± 0.000207	0.051 ± 0.001	1870.1 ± 16.5	1.01 ± 0.01	3.25 ± 0.77
5	28	96	0.001612 ± 0.000013	0.001810 ± 0.000010	0.000085 ± 0.000001	3.247778 ± 0.000111	0.053 ± 0.001	2015.0 ± 16.8	1.12 ± 0.01	3.95 ± 0.78
6	38	134	0.001271 ± 0.000011	0.001701 ± 0.000008	0.000077 ± 0.000001	3.338897 ± 0.000271	0.060 ± 0.001	2627.7 ± 22.1	1.34 ± 0.01	2.86 ± 0.86
7	100	234	0.000332 ± 0.000003	0.000883 ± 0.000004	0.000065 ± 0.000001	1.378120 ± 0.000213	0.197 ± 0.004	4152.7 ± 36.7	2.66 ± 0.03	3.06 ± 1.10
8	300	534	0.001191 ± 0.000010	0.003008 ± 0.000014	0.000266 ± 0.000002	4.503675 ± 0.000546	0.223 ± 0.002	3780.3 ± 31.6	2.52 ± 0.02	2.91 ± 0.43
9	500	1034	0.000929 ± 0.000008	0.002335 ± 0.000012	0.000500 ± 0.000002	3.617411 ± 0.001842	0.538 ± 0.005	3894.6 ± 33.7	2.51 ± 0.03	1.56 ± 0.18
10	700	1734	0.000780 ± 0.000007	0.001954 ± 0.000009	0.000682 ± 0.000003	3.018096 ± 0.000507	0.873 ± 0.009	3867.0 ± 33.1	2.50 ± 0.02	1.03 ± 0.14
11	850	2584	0.000496 ± 0.000004	0.001212 ± 0.000007	0.000711 ± 0.000003	2.026010 ± 0.000334	1.434 ± 0.014	4084.9 ± 36.8	2.44 ± 0.03	0.87 ± 0.13
12	1000	3584	0.000334 ± 0.000003	0.000791 ± 0.000004	0.000676 ± 0.000003	1.323481 ± 0.000277	2.027 ± 0.021	3967.0 ± 36.3	2.37 ± 0.02	0.76 ± 0.15
13	1000	4584	0.000220 ± 0.000002	0.000488 ± 0.000003	0.000533 ± 0.000002	0.884701 ± 0.000263	2.428 ± 0.026	4029.1 ± 40.0	2.22 ± 0.03	0.62 ± 0.15
14	1000	5584	0.000150 ± 0.000001	0.000309 ± 0.000002	0.000420 ± 0.000002	0.581805 ± 0.000168	2.790 ± 0.030	3868.7 ± 36.1	2.05 ± 0.02	0.54 ± 0.15
15	1000	6584	0.000118 ± 0.000001	0.000230 ± 0.000002	0.000319 ± 0.000002	0.461247 ± 0.000101	2.700 ± 0.033	3900.8 ± 42.5	1.95 ± 0.03	0.63 ± 0.19
16	1000	7584	0.000099 ± 0.000001	0.000196 ± 0.000002	0.000256 ± 0.000002	0.376979 ± 0.000067	2.581 ± 0.033	3804.1 ± 43.3	1.98 ± 0.03	0.35 ± 0.30
17	1000	8584	0.000077 ± 0.000001	0.000158 ± 0.000001	0.000229 ± 0.000003	0.305095 ± 0.000083	2.958 ± 0.042	3949.8 ± 34.2	2.05 ± 0.02	0.20 ± 0.44
18	1000	9584	0.000064 ± 0.000001	0.000125 ± 0.000001	0.000190 ± 0.000001	0.248347 ± 0.000034	2.957 ± 0.039	3861.4 ± 43.3	1.95 ± 0.03	0.27 ± 0.38
19	1000	10584	0.000052 ± 0.000001	0.000103 ± 0.000001	0.000180 ± 0.000001	0.219229 ± 0.000036	3.495 ± 0.049	4253.0 ± 52.7	2.00 ± 0.03	0.01 ± 0.36
20	1000	11584	0.000052 ± 0.000001	0.000092 ± 0.000001	0.000162 ± 0.000001	0.207476 ± 0.000034	3.112 ± 0.043	3989.5 ± 52.6	1.78 ± 0.03	0.01 ± 0.35
The argon isotopes are listed in volt, *J* value: 0.0083542 ± 0.0000418, sample weight: 151.6 mg										
GU91										
1	24	24	0.000045 ± 0.000001	0.000163 ± 0.000001	0.000024 ± 0.000000	0.171275 ± 0.000043	0.536 ± 0.013	3806.3 ± 54.0	3.63 ± 0.06	1.99 ± 2.53
2	100	124	0.000190 ± 0.000002	0.000698 ± 0.000003	0.000090 ± 0.000001	0.600037 ± 0.000065	0.472 ± 0.006	3154.8 ± 28.4	3.67 ± 0.04	2.41 ± 0.66
3	150	274	0.000284 ± 0.000002	0.000951 ± 0.000005	0.000139 ± 0.000001	1.054335 ± 0.000131	0.491 ± 0.006	3716.5 ± 30.9	3.35 ± 0.03	1.05 ± 0.51
4	200	474	0.000249 ± 0.000002	0.000930 ± 0.000004	0.000181 ± 0.000001	0.894927 ± 0.000122	0.728 ± 0.008	3591.7 ± 32.6	3.73 ± 0.04	0.08 ± 0.36
5	250	724	0.000217 ± 0.000002	0.000791 ± 0.000004	0.000220 ± 0.000001	0.798887 ± 0.000052	1.016 ± 0.011	3681.9 ± 33.2	3.65 ± 0.04	0.15 ± 0.28
6	300	1024	0.000216 ± 0.000002	0.000751 ± 0.000004	0.000262 ± 0.000001	0.744899 ± 0.000061	1.212 ± 0.012	3447.0 ± 31.8	3.47 ± 0.04	0.17 ± 0.26
7	350	1374	0.000191 ± 0.000002	0.000700 ± 0.000003	0.000296 ± 0.000001	0.733718 ± 0.000065	1.549 ± 0.016	3834.4 ± 34.8	3.66 ± 0.04	0.16 ± 0.24
8	400	1774	0.000165 ± 0.000002	0.000597 ± 0.000003	0.000329 ± 0.000002	0.632588 ± 0.000083	1.995 ± 0.023	3837.8 ± 40.3	3.62 ± 0.04	0.35 ± 0.18
9	450	2224	0.000118 ± 0.000001	0.000406 ± 0.000002	0.000330 ± 0.000002	0.477897 ± 0.000049	2.800 ± 0.030	4056.1 ± 38.6	3.44 ± 0.04	0.05 ± 0.18
10	500	2724	0.000097 ± 0.000001	0.000289 ± 0.000001	0.000341 ± 0.000001	0.384512 ± 0.000115	3.528 ± 0.038	3977.4 ± 41.2	2.99 ± 0.03	0.66 ± 0.19
11	600	3324	0.000102 ± 0.000001	0.000285 ± 0.000002	0.000366 ± 0.000002	0.385941 ± 0.000133	3.586 ± 0.038	3780.0 ± 37.2	2.79 ± 0.03	0.28 ± 0.23
12	700	4024	0.000108 ± 0.000001	0.000320 ± 0.000002	0.000384 ± 0.000002	0.425840 ± 0.000137	3.546 ± 0.035	3931.3 ± 33.8	2.96 ± 0.03	0.77 ± 0.22
13	800	4824	0.000102 ± 0.000001	0.000304 ± 0.000002	0.000384 ± 0.000001	0.383869 ± 0.000151	3.767 ± 0.037	3762.8 ± 34.0	2.98 ± 0.03	0.69 ± 0.28
14	1033	5857	0.000105 ± 0.000001	0.000306 ± 0.000002	0.000418 ± 0.000002	0.420899 ± 0.000157	3.974 ± 0.047	3998.8 ± 44.5	2.91 ± 0.04	0.97 ± 0.26
15	1100	6957	0.000089 ± 0.000001	0.000251 ± 0.000002	0.000382 ± 0.000002	0.358164 ± 0.000126	4.313 ± 0.048	4039.1 ± 37.9	2.83 ± 0.03	0.70 ± 0.24
16	1200	8157	0.000080 ± 0.000001	0.000207 ± 0.000001	0.000359 ± 0.000002	0.299037 ± 0.000094	4.482 ± 0.052	3734.0 ± 40.2	2.58 ± 0.03	0.65 ± 0.25
17	1300	9457	0.000068 ± 0.000001	0.000170 ± 0.000001	0.000336 ± 0.000002	0.257509 ± 0.000114	4.944 ± 0.055	3789.7 ± 37.7	2.50 ± 0.03	0.86 ± 0.25
18	1400	10857	0.000058 ± 0.000001	0.000139 ± 0.000001	0.000323 ± 0.000002	0.216585 ± 0.000100	5.574 ± 0.094	3738.6 ± 57.3	2.39 ± 0.04	1.37 ± 0.27
19	1500	12357	0.000050 ± 0.000001	0.000112 ± 0.000001	0.000318 ± 0.000002	0.191670 ± 0.000090	6.347 ± 0.103	3824.4 ± 56.6	2.24 ± 0.04	0.88 ± 0.31
The argon isotopes are listed in volt, *J* value: 0.0090085 ± 0.0000450, sample weight: 151.0 mg										
GU103-1										
1	30	30	0.000159 ± 0.000002	0.000106 ± 0.000001	0.000017 ± 0.000000	0.088484 ± 0.000031	0.106 ± 0.003	556.1 ± 5.4	0.66 ± 0.01	4.54 ± 2.41
2	100	130	0.000328 ± 0.000003	0.000360 ± 0.000002	0.000056 ± 0.000001	0.232279 ± 0.000049	0.172 ± 0.002	709.2 ± 6.8	1.10 ± 0.01	1.33 ± 0.79
3	150	280	0.000323 ± 0.000003	0.000456 ± 0.000003	0.000084 ± 0.000001	0.269304 ± 0.000029	0.259 ± 0.003	833.1 ± 7.5	1.41 ± 0.02	0.54 ± 0.63
4	200	480	0.000231 ± 0.000002	0.000399 ± 0.000002	0.000101 ± 0.000001	0.229112 ± 0.000027	0.435 ± 0.006	990.0 ± 9.1	1.72 ± 0.02	0.63 ± 0.55
5	300	780	0.000244 ± 0.000002	0.000514 ± 0.000002	0.000154 ± 0.000001	0.286290 ± 0.000064	0.630 ± 0.006	1172.1 ± 10.8	2.10 ± 0.02	0.24 ± 0.50
6	400	1180	0.000248 ± 0.000002	0.000637 ± 0.000003	0.000205 ± 0.000001	0.347304 ± 0.000040	0.827 ± 0.009	1400.0 ± 12.7	2.57 ± 0.03	0.16 ± 0.43
7	500	1680	0.000168 ± 0.000002	0.000516 ± 0.000003	0.000237 ± 0.000001	0.291672 ± 0.000039	1.405 ± 0.016	1732.5 ± 17.7	3.06 ± 0.03	0.51 ± 0.21
8	600	2280	0.000125 ± 0.000001	0.000397 ± 0.000002	0.000261 ± 0.000001	0.251037 ± 0.000024	2.085 ± 0.024	2003.6 ± 21.4	3.17 ± 0.04	0.67 ± 0.16
9	700	2980	0.000098 ± 0.000001	0.000303 ± 0.000002	0.000249 ± 0.000001	0.213500 ± 0.000041	2.535 ± 0.029	2176.2 ± 22.8	3.09 ± 0.04	0.85 ± 0.22
10	850	3830	0.000090 ± 0.000001	0.000240 ± 0.000002	0.000243 ± 0.000001	0.192803 ± 0.000047	2.697 ± 0.030	2139.1 ± 22.2	2.66 ± 0.03	0.64 ± 1.39
11	1150	4980	0.000082 ± 0.000001	0.000192 ± 0.000001	0.000260 ± 0.000002	0.183953 ± 0.000026	3.150 ± 0.041	2230.3 ± 24.4	2.33 ± 0.03	1.06 ± 1.41
12	1000	5980	0.000055 ± 0.000001	0.000117 ± 0.000001	0.000178 ± 0.000001	0.120868 ± 0.000021	3.240 ± 0.052	2206.0 ± 32.2	2.13 ± 0.03	2.08 ± 1.86
13	1000	6980	0.000045 ± 0.000001	0.000095 ± 0.000001	0.000153 ± 0.000001	0.102982 ± 0.000024	3.424 ± 0.070	2300.8 ± 43.5	2.12 ± 0.05	1.46 ± 2.17
14	1100	8080	0.000039 ± 0.000000	0.000075 ± 0.000001	0.000141 ± 0.000001	0.090452 ± 0.000014	3.603 ± 0.049	2303.2 ± 28.4	1.91 ± 0.03	0.53 ± 1.65
15	1300	9380	0.000039 ± 0.000001	0.000076 ± 0.000001	0.000153 ± 0.000001	0.095419 ± 0.000021	3.918 ± 0.072	2450.2 ± 44.3	1.95 ± 0.04	0.53 ± 1.65
16	1500	10880	0.000039 ± 0.000001	0.000070 ± 0.000001	0.000158 ± 0.000001	0.095212 ± 0.000017	4.097 ± 0.079	2461.2 ± 44.4	1.81 ± 0.04	0.70 ± 0.55
The argon isotopes are listed in volt, *J* value: 0.0088236 ± 0.0000441, sample weight: 153.7 mg										
GU103-2										
1	7	7	0.000007 ± 0.000000	0.000041 ± 0.000000	0.000006 ± 0.000001	0.019917 ± 0.000025	0.896 ± 0.098	2948.7 ± 81.1	6.05 ± 0.18	
2	200	207	0.000190 ± 0.000002	0.000947 ± 0.000005	0.000184 ± 0.000001	0.464624 ± 0.000049	0.970 ± 0.012	2448.5 ± 24.5	4.99 ± 0.06	
3	250	457	0.000161 ± 0.000001	0.000809 ± 0.000004	0.000219 ± 0.000001	0.417376 ± 0.000043	1.362 ± 0.015	2594.0 ± 24.1	5.02 ± 0.05	
4	300	757	0.000172 ± 0.000002	0.000834 ± 0.000004	0.000262 ± 0.000002	0.441730 ± 0.000049	1.526 ± 0.016	2571.4 ± 22.5	4.85 ± 0.05	0.11 ± 0.28
5	350	1107	0.000171 ± 0.000002	0.000815 ± 0.000004	0.000310 ± 0.000001	0.458500 ± 0.000057	1.809 ± 0.019	2678.0 ± 26.5	4.76 ± 0.05	0.02 ± 0.21
6	400	1507	0.000172 ± 0.000002	0.000780 ± 0.000004	0.000345 ± 0.000002	0.458102 ± 0.000077	2.006 ± 0.023	2664.0 ± 25.4	4.54 ± 0.05	0.30 ± 0.21
7	450	1957	0.000138 ± 0.000001	0.000643 ± 0.000004	0.000332 ± 0.000001	0.393903 ± 0.000610	2.399 ± 0.025	2844.3 ± 27.2	4.65 ± 0.05	0.72 ± 0.23
8	500	2457	0.000131 ± 0.000001	0.000592 ± 0.000003	0.000340 ± 0.000002	0.371757 ± 0.000040	2.600 ± 0.032	2839.0 ± 30.7	4.52 ± 0.05	1.31 ± 0.23
9	550	3007	0.000109 ± 0.000001	0.000464 ± 0.000002	0.000324 ± 0.000002	0.318664 ± 0.000049	2.981 ± 0.038	2933.2 ± 32.9	4.27 ± 0.05	1.03 ± 0.20
10	600	3607	0.000095 ± 0.000001	0.000396 ± 0.000002	0.000321 ± 0.000002	0.290484 ± 0.000032	3.370 ± 0.039	3053.7 ± 32.5	4.16 ± 0.05	1.56 ± 0.24
11	650	4257	0.000079 ± 0.000001	0.000307 ± 0.000002	0.000299 ± 0.000001	0.248361 ± 0.000020	3.802 ± 0.044	3163.3 ± 33.3	3.92 ± 0.05	1.69 ± 0.26
12	700	4957	0.000063 ± 0.000001	0.000229 ± 0.000001	0.000263 ± 0.000001	0.204508 ± 0.000027	4.153 ± 0.044	3228.1 ± 32.9	3.62 ± 0.04	1.86 ± 0.24
13	750	5707	0.000060 ± 0.000001	0.000193 ± 0.000001	0.000244 ± 0.000001	0.190622 ± 0.000043	4.041 ± 0.039	3156.7 ± 28.4	3.19 ± 0.03	1.84 ± 0.27
14	800	6507	0.000049 ± 0.000001	0.000155 ± 0.000001	0.000219 ± 0.000001	0.158863 ± 0.000029	4.444 ± 0.059	3219.5 ± 39.3	3.14 ± 0.04	2.21 ± 0.32
15	850	7357	0.000044 ± 0.000000	0.000143 ± 0.000001	0.000195 ± 0.000001	0.144272 ± 0.000019	4.432 ± 0.041	3275.5 ± 27.7	3.26 ± 0.03	2.77 ± 0.52
16	900	8257	0.000044 ± 0.000001	0.000142 ± 0.000001	0.000183 ± 0.000001	0.138010 ± 0.000025	4.170 ± 0.052	3150.1 ± 36.3	3.24 ± 0.04	2.34 ± 0.38
17	1000	9257	0.000042 ± 0.000000	0.000140 ± 0.000001	0.000188 ± 0.000001	0.136838 ± 0.000042	4.439 ± 0.058	3237.4 ± 38.1	3.30 ± 0.04	2.92 ± 0.50
18	1400	10657	0.000052 ± 0.000001	0.000167 ± 0.000001	0.000235 ± 0.000001	0.170483 ± 0.000043	4.562 ± 0.050	3307.5 ± 34.8	3.24 ± 0.04	2.95 ± 0.55
19	1400	12057	0.000045 ± 0.000000	0.000145 ± 0.000001	0.000221 ± 0.000001	0.153526 ± 0.000014	4.868 ± 0.056	3388.4 ± 36.0	3.19 ± 0.04	2.79 ± 0.37
The argon isotopes are listed in volt, *J* value: 0.0085909 ± 0.0000430, sample weight: 154.0 mg										

**Figure 6.**
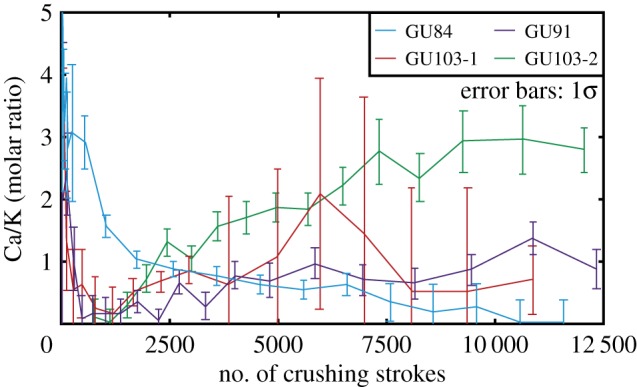
Degassing patterns of Ca/K calculated by irradiated argon isotopes via *in vacuo* stepwise crushing. Ca-rich fluid inclusions were extracted in the early crushing steps. Large Ca/K variation from approximately 0 to 3 among samples in later steps.

The releasing patterns of Ca/K calculated by argon isotopes show that high Ca/K value was extracted in the early steps and that various Ca/K value among samples was extracted in the later steps (figure 6). GU84 shows K enrichment in the later steps, whereas the others show K enrichment in the middle steps and Ca enrichment in the later steps. In the Ca/K value, apparent characteristics that were observed for all samples were absent except Ca enrichment in early steps.

The K-rich components extracted by crushing probably results from the smaller K-rich fluid inclusions. However, the specific release pattern of ^39^Ar_K_ makes it possible to consider the contribution from K-bearing impurity minerals in the hydrothermal quartz. Indeed, the presence of muscovite inclusions was identified in the quartz crystals, as described above, which is consistent with the geochemical study of the Ongeluk lavas that revealed that the pillow rims and hyaloclastite are enriched in K as a result of the seafloor hydrothermal alteration [33]. Furthermore, argon gas derived from the irradiated K-bearing minerals, such as mica, muscovite and scapolite, can also generally be extracted via the crushing technique [54,55]. However, the high Ca/K compositions in the later steps of GU91, GU103-1 and GU103-2 are inconsistent with the contribution of K-rich minerals (figure 6).

In the early steps, the amount of ^39^Ar_K_ gas derived from smaller K-rich inclusions is small or negligible because of a large amount of argon gas mainly extracted from larger fluid inclusions containing most of ^36^Ar_A_, ^38^Ar_Cl_ and ^40^Ar_A+R+E_. Especially during the crushing extraction of GU84 and GU103-1, the most dominant argon isotope changed from ^36^Ar_A_ to ^38^Ar_Cl_ in the early steps, indicating that the isotope ratio of the released argon changed as the number of steps increased. Therefore, the samples GU84 and GU103-1 should contain at least two types of fluid inclusions with different argon isotope ratios in the early steps (see details below). In the middle to later steps, the contributions of ^39^Ar_K_ from K-rich smaller fluid inclusions become significant due to the decreasing amount of argon released from the larger fluid inclusions as the crushing proceeded (table 1; figure 5). Therefore, the obtained argon isotopic composition is probably formed by three-component mixing in each sample during crushing extraction.

### 5.2. Isotopic compositions of each sample

**Figure 7.**
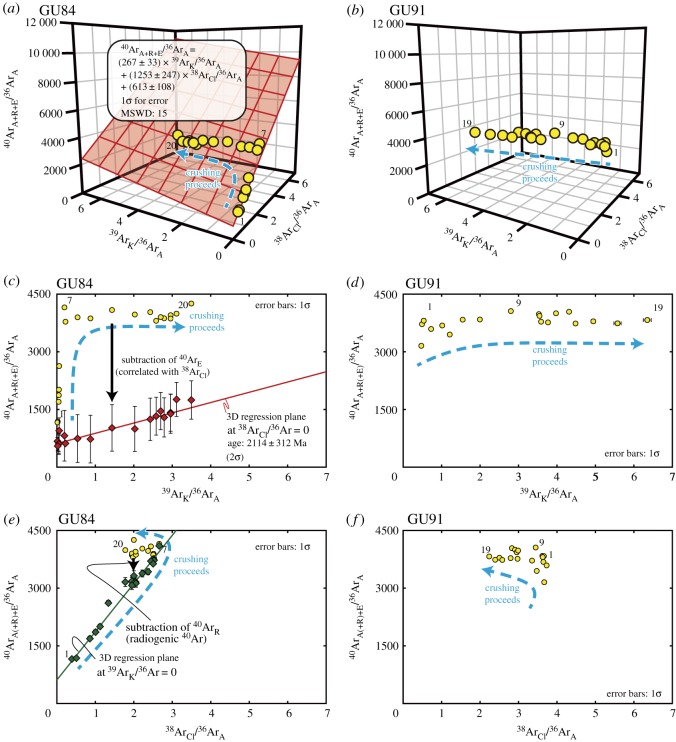
Isotopic compositions of argon measured for GU84 and GU91. Argon isotope ratios of (*a*) GU84 and (*b*) GU91 are plotted in the 3D ^40^Ar_A+R+E_/^36^Ar_A_–^39^Ar_K_/^36^Ar_A_–^38^Ar_Cl_/^36^Ar_A_ space (yellow spheres). A blue dashed arrow indicates the crushing progress, while the numbers of the representative steps are near the spheres. The obtained 3D regression plane for GU84 is shown as a red plane in (*a*); the isotopic composition of GU91 in (*b*) shows a linear distribution in this space, which prevents fitting on the 3D regression plane. (*c*) ^40^Ar_E_-corrected and -uncorrected argon isotope ratios (red diamond and yellow circle, respectively) of GU84 plotted on a 2D isochron diagram. The ^40^Ar_E_-corrected isotope ratios show a linear mixing trend between the atmospheric component and the radiogenic component derived from K-rich fluid inclusions or K-rich minerals. (*d*) Isotopic composition of GU91 plotted on a 2D isochron diagram showing constant ^40^Ar_A+R+E_/^36^Ar_A_ values with a large variation of ^39^Ar_K_/^36^Ar_A_. (*e*) ^40^Ar_R_-corrected and -uncorrected isotope ratios of GU84 (green diamond and yellow circle, respectively) plotted on ^40^Ar_A(+R)+E_/^36^Ar_A_ versus ^38^Ar_Cl_/^36^Ar_A_ diagram showing a linear mixing trend between the atmospheric component and the ^40^Ar_E_-rich component. (*f*) Isotope ratios of GU91 plotted on an ^40^Ar_A+R+E_/^36^Ar_A_ versus ^38^Ar_Cl_/^36^Ar_A_ diagram showing no apparent correlation.

**Figure 8.**
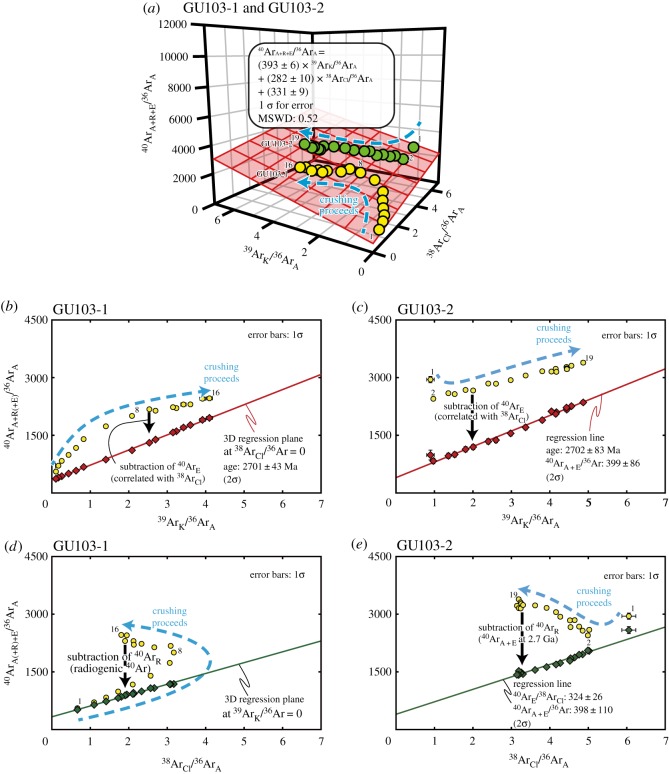
(*a*) Isotopic compositions of argon for GU103-1 and GU103-2 plotted in the 3D ^40^Ar_A+R+E_/^36^Ar_A_–^39^Ar_K_/^36^Ar_A_–^38^Ar_Cl_/^36^Ar_A_ space. The isotope ratios of GU103-1 show a planar distribution in the 3D space, while the isotope ratios of GU103-2 show a linear distribution along the plane of GU103-1. (*b,c*) ^40^Ar_E_-corrected and -uncorrected argon isotope ratios plotted on a 2D isochron diagram for GU103-1 and GU103-2, respectively, showing a linear mixing trend between the atmospheric component and the radiogenic component from K-rich fluid inclusions or K-rich minerals. (*d,e*) ^40^Ar_R_-corrected and -uncorrected isotope ratios of GU103-1 and GU103-2, respectively, plotted on an ^40^Ar_A(+R)+E_/^36^Ar_A_ versus ^38^Ar_Cl_/^36^Ar_A_ diagram showing a linear mixing trend between the atmospheric component and the ^40^Ar_E_-rich component. ^40^Ar_E_-corrected value and ^40^Ar_R_-corrected value of GU103-2 are additionally corrected by the difference of *J* value between GU103-1 and Gu103-2. All symbols in this figure correspond to those in figure 7.

Based on the obtained isotopic data, the argon gas from each sample is thought to be a mixture of at least three independent components derived from irradiated three types of fluid inclusions. Because a three-component dataset should distribute along a single plane in a 3D plot, the results were plotted in 3D ^40^Ar_A+R+E_/^36^Ar_A_–^39^Ar_K_/^36^Ar_A_–^38^Ar_Cl_/^36^Ar_A_ space (figures 7 and 8). Two (GU84, GU103) of the three samples show planar correlations in this 3D spaces calculated by Isoplot 4.15 [56].

In previous studies, a correlation between ^38^Ar_Cl_ and ^40^Ar_A+R+E_ has been observed for argon isotopes extracted from fluid inclusions via the crushing technique (e.g. [19]); however, ^39^Ar_K_ has not sufficiently been extracted in the crushing experiments, instead it has been extracted from using heating experiments. The distinct behaviour of ^39^Ar_K_ with different extraction methods indicates that the ^39^Ar_K_ extracted via the crushing method was derived from fluid inclusions while that extracted via the heating method originated from minerals (e.g. [19]). Despite the different origins of argon isotopes, some of their datasets show planar distributions, which were interpreted as mixing planes between the three representative components: the atmospheric component (^40^Ar_A_ and ^36^Ar_A_) in fluid inclusions, the excess Ar-rich component (^38^Ar_Cl_ and Cl-correlated ^40^Ar as ^40^Ar_E_) in fluid inclusions and the radiogenic Ar component (^39^Ar_K_ and ^39^Ar_K_-correlated ^40^Ar as ^40^Ar_R_) in K-bearing minerals [19,20,22,57].

Assuming that the planar correlations obtained in this study were also generated by the mechanism similar to mixing plane from heating and crushing experiment, the obtained 3D regression planes enable us to calculate ^40^Ar_E_ and ^40^Ar_R_. Therefore, the Ar–Ar age can be calculated from the ^40^Ar_R_/^39^Ar_K_ values. Furthermore, the origin of the fluid can also be constrained because the subtraction of the ^40^Ar_R_/^36^Ar_A_ values from the ^40^Ar_A+R+E_/^36^Ar_A_ values can be used to estimate the degree of mixing between the atmospheric component and the ^40^Ar_E_-rich component (figures 7*e,f* and 8*d,e*). We describe the results of the isotopic data in detail for each sample below.

#### 5.2.1. GU84

The sample GU84 exhibits positive correlations of ^40^Ar_A+R+E_/^36^Ar_A_ to ^38^Ar_Cl_/^36^Ar_A_, and ^39^Ar_K_/^36^Ar_A_ and from step 1 to 7 corresponding to from 8 to 234 strokes (table 1; figure 7*c,e*). Then, from step 8 to 20 (from 534 to 11 584 strokes), the sample shows a decrease in ^38^Ar_Cl_/^36^Ar_A_, an increase in ^39^Ar_K_/^36^Ar_A_ and a relatively constant value of ^40^Ar_A+R+E_/^36^Ar_A_. The 3D plot of the argon isotopes shows strong correlations between ^38^Ar_Cl_/^36^Ar_A_, ^39^Ar_K_/^36^Ar_A_ and ^40^Ar_A+R+E_/^36^Ar_A_ with the intercept of ^40^Ar_A+E_/^36^Ar_A_ = 613 ± 108 (1*σ*). The intercept value is significantly higher than the modern atmospheric value (298.6; [58]) (figure 7*a*). The ^40^Ar_A+R_/^36^Ar_A_ value without the contribution of ^40^Ar_E_, equivalent to the subtraction of ^40^Ar_E_/^36^Ar_A_ from ^40^Ar_A+R+E_/^36^Ar_A_, shows a linear positive correlation with ^39^Ar_K_/^36^Ar_A_ (figure 7*c*). However, the corrected value also shows a large uncertainty due to the relatively large uncertainty in the 3D regression plane while the mean square weighted deviation (MSWD) value for the sample is 15. This 3D regression plane gives an age of 2114 ± 312 Ma (±2*σ*). The ^40^Ar_A+E_/^36^Ar_A_ value shows a slightly scattered but linear positive correlation with ^38^Ar_Cl_/^36^Ar_A_ (figure 7*e*). The highest value of ^40^Ar_A+E_/^36^Ar_A_ (equivalent to the subtraction of ^40^Ar_R_/^36^Ar_A_ from ^40^Ar_A+R+E_/^36^Ar_A_) is 4100, which can be used to interpret the origin of the fluids.

The large MSWD value clearly indicates that the uncertainty in the 3D model for GU84 is insufficient to obtain a precise age. Especially, in the early extraction steps of GU84, the scattered argon isotope ratios generated a large uncertainty in the 3D regression plane (figure 7*c,e*). Previously, it has been reported that degassing from ^40^Ar_E_-rich secondary fluid inclusions in the early extraction steps can cause scattered argon isotope ratios (e.g. [59]). Such trends potentially account for the relatively large variations in the argon isotope ratios in the early steps of the extraction of GU84.

#### 5.2.2. GU91

The sample GU91 exhibits increases in ^39^Ar_K_/^36^Ar_A_ and ^40^Ar_A+R+E_/^36^Ar_A_ and a decrease in ^38^Ar_Cl_/^36^Ar_A_ from step 1 to 9 (from 24 to 2224 strokes) on the two-dimensional (2D) diagrams (table 1; figure 7*d,f*). Then, the sample shows a decrease in ^38^Ar_Cl_/^36^Ar_A_, an increase in ^39^Ar_K_/^36^Ar_A_ and relatively constant values of ^40^Ar_A+R+E_/^36^Ar_A_ from step 10 to 19 (from 2724 to 12 357 strokes). The dataset of GU91 could not be fitted to a 3D regression plane due to its relatively linear distribution in 3D space. The largest uncorrected ^40^Ar_A+R+E_/^36^Ar_A_ value is 4056.

#### 5.2.3. GU103-1

The sample GU103-1 exhibits positive correlations between ^38^Ar_Cl_/^36^Ar_A_, ^39^Ar_K_/^36^Ar_A_ and ^40^Ar_A+R+E_/^36^Ar_A_ from step 1 to 8 (from 30 to 2280 strokes) on the 2D diagrams (table 1; figure 8*b,d*). Then, the isotopic compositions show a decrease in ^38^Ar_Cl_/^36^Ar_A_ and increases in ^39^Ar_K_/^36^Ar_A_ and ^40^Ar_A+R+E_/^36^Ar_A_ from step 9 to 16 (from 2980 to 10 880 strokes). The isotopic compositions plotted in the 3D space show strong correlations between ^38^Ar_Cl_/^36^Ar_A_, ^39^Ar_K_/^36^Ar_A_ and ^40^Ar_A+R+E_/^36^Ar_A_ with the intercept of ^40^Ar_A+E_/^36^Ar_A_ = 331 ± 9 (1*σ*), which is close to the atmospheric value (figure 8*a*). ^40^Ar_A+R_/^36^Ar_A_ shows an excellent linear positive correlation with the ^39^Ar_K_/^36^Ar_A_ values (figure 8*c*) while the MSWD value of the 3D planar regression is 0.52. The obtained regression plane gives an age of 2701 ± 43 Ma (2*σ*). The ^40^Ar_R_-corrected ^40^Ar_A+E_/^36^Ar_A_ values show a linear positive correlation with the ^38^Ar_Cl_/^36^Ar_A_ values (figure 8*d*). The highest value of ^40^Ar_A+E_/^36^Ar_A_ is 1142. The well-constrained regression plane and the low MSWD value in this experiment indicate that the argon isotope ratios of GU103-1 can be satisfactorily explained by the 3D model and are sufficiently suitable for a discussion of the origin and age of the Ongeluk hydrothermal quartz and its fluid inclusions.

#### 5.2.4. GU103-2

The results of the extraction from GU103-2 also exhibit relatively good correlations between ^38^Ar_Cl_/^36^Ar_A_, ^39^Ar_K_/^36^Ar_A_ and ^40^Ar_A+R+E_/^36^Ar_A_ except for step 1 (7 strokes) on the 2D diagrams (figure 8*c,e*). The isotopic compositions show a decrease in ^38^Ar_Cl_/^36^Ar_A_ and increases in ^39^Ar_K_/^36^Ar_A_ and ^40^Ar_A+R+E_/^36^Ar_A_ from step 2 to 19 (from 207 to 12 057 strokes). The isotopic compositions plotted in the 3D space show correlations between ^38^Ar_Cl_/^36^Ar_A_, ^39^Ar_K_/^36^Ar_A_ and ^40^Ar/^36^Ar_A_ and the distribution along the regression plane of GU103-1 (figure 8*a*). The difference between GU103-1 and GU103-2 results from the contribution of the atmospheric component and the *J* value. Therefore, the result of GU103-2 can be treated the same as the data from GU103-1. An age correction of the same age as GU103-1 to the GU103-2 data provides a similar regression line on a diagram of between ^38^Ar_Cl_/^36^Ar_A_ and ^40^Ar_A+E_/^36^Ar. The intercept ^40^Ar_A+E_/^36^Ar_A_ value of the regression lines on the ^38^Ar_Cl_/^36^Ar_A_ and ^40^Ar_A+E_/^36^Ar_A_ diagram and isochron diagram are 398 ± 55 (1*σ*) and 399 ± 43 (1*σ*), respectively. The highest ^40^Ar_A+E_/^36^Ar_A_ value is 2586.

#### 5.2.5. Consistency between the 3D model and argon isotope ratios

**Figure 9.**
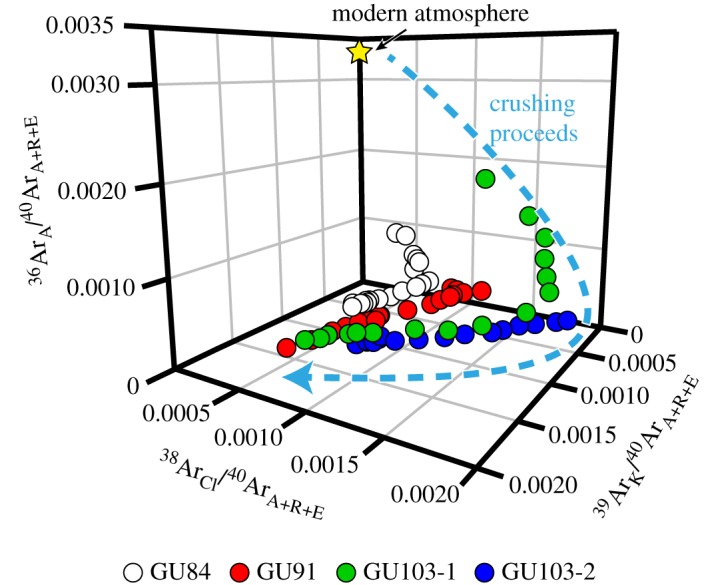
Isotopic compositions of argon for all samples plotted in the 3D ^36^Ar_A_/^40^Ar_A+R+E_–^39^Ar_K_/^40^Ar_A+R+E_–^38^Ar_Cl_/^40^Ar_A+R+E_ space. The isotope ratios of all samples show the composition changes from atmospheric-rich composition to K-rich composition through Cl-rich composition.

The argon isotope ratios obtained for the fluid inclusions and minerals are generally explained as mixing between atmospheric argon, radiogenic argon and excess argon when the values can be plotted on a 3D regression plane (e.g. [57,60]). Even though GU84 have relatively large uncertainties resulting from the scattered argon isotopic ratio, the results of GU103-1 show a well-constrained 3D planar distribution with the intercept value (331 ± 9, 1*σ*) close to atmospheric ^40^Ar_A_/^36^Ar_A_ of 298.6. The 3D plot of argon isotopes normalized by ^40^Ar_A+R+E_ also shows the change in the composition as crushing proceeds from atmospheric endmember to K-rich endmember through Cl-rich endmember (figure 9). Therefore, the 3D distribution of GU103-1 probably resulted from mixing between atmospheric argon (EM1), ^40^Ar_E_- and Cl-rich fluid (EM2) and radiogenic argon from K-rich fluid (EM3).

**Figure 10.**
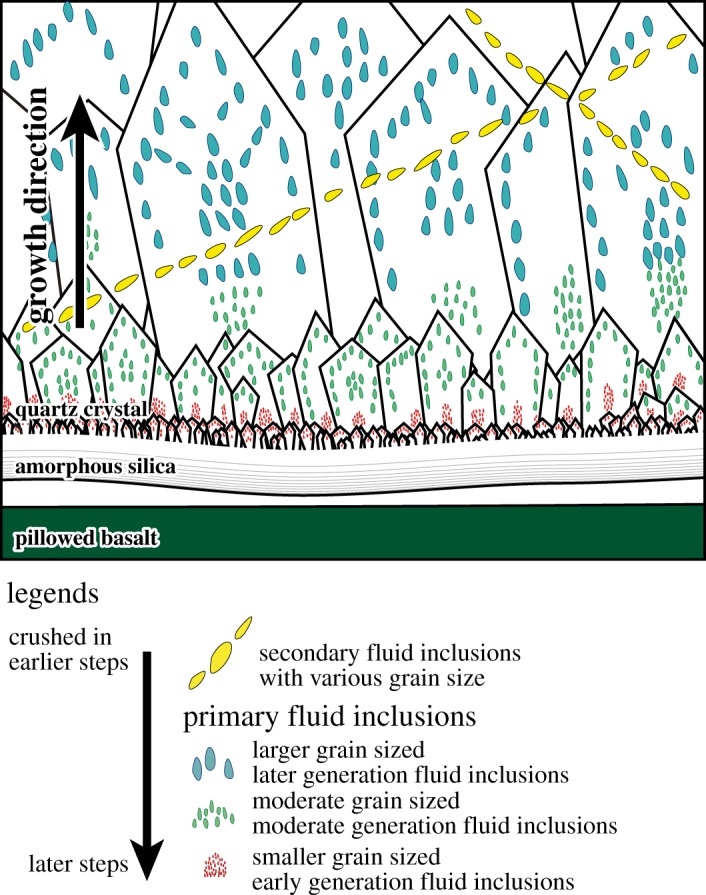
Schematic image of the fluid inclusions in the hydrothermal quartz, showing an interpretation based on the release patterns of argon gas via crushing and the argon isotopic compositions. Secondary and larger primary fluid inclusions that formed later are crushed in earlier steps while smaller ones are crushed in later steps.

The results of the isotope ratio show a continuous change in the composition as the crushing proceeds. This change probably resulted from the change in the generation of the crushed fluid inclusions from the later formed larger inclusions to the earlier formed smaller inclusions (figure 10). In previous studies using a similar crushing method for argon extraction, excess argon was extracted from secondary fluid inclusions, which are easily broken by crushing, before the extraction of the excess argon-free composition from the primary fluid inclusions (e.g. [61–63]). However, in this study, small amounts of excess argon are extracted in early steps before the degassing of significant amounts of excess argon in the later steps. This indicates that the primary fluid inclusions are composed of excess argon. Another characteristic of this study compared to previous studies is the remaining contribution of fluid inclusions primarily crushed in the early steps through all the crushing steps. The difference between the result of GU103-1 and that of GU103-2 can be explained via the different contributions of EM1, supporting the remaining contribution of EM1. The degassing pattern of EM1 suggests that the change in the composition as crushing proceeds resulted from the difference in the size distributions of each generation of fluid inclusion rather than from the different physical strength of the primary and secondary inclusions. Therefore, all endmembers of the fluid inclusions are probably composed of primary fluid inclusions (figure 10).

### 5.3. Interpretation of quartz formation age

#### 5.3.1. Interpretation of obtained ages

The stepwise crushing experiments for the Ar–Ar dating provided ages of 2114 ± 312 Ma (2*σ*) for GU84 and 2701 ± 43 Ma (2*σ*) for GU103-1. The analysed quartz samples should ideally have similar ages because these samples were collected from outcrops in the same locality even though the obtained ages vary from 2114 ± 312 Ma for GU84 to 2701 ± 43 Ma for GU103-1.

There are several effects that can potentially make the Ar–Ar age younger than the true age. One possibility is a break in the closed system for argon, such as the recrystallization of quartz. However, quartz retains its microcrystalline texture (figure 3*c,e*), indicating the absence of significant recrystallization and any resulting escape of argon. A second possibility is mixing between the argon from K-rich inclusion and the excess argon without a correlation with chlorine from the (secondary) fluid inclusions. This effect may have influenced all the samples of GU84; especially in the early steps of GU84 (figure 7*c*), it may have disturbed the obtained ages and intercept value of ^40^Ar_A+E_/^36^Ar_A_ for each sample.

Nevertheless, the sample GU103-1 is probably unaffected by this effect because the 3D distribution of argon isotopes is in good agreement with the three-component mixing model. Based on this consistency, 2701 ± 43 Ma for GU103-1 is the best constrained age. However, this age is significantly older than the eruption age of the Ongeluk lavas (2426 ± 3 Ma); even the ages of igneous and sedimentary rocks vary from 2642 ± 3 to 1928 ± 4 Ma in the Griqualand West Basin. Such a discrepancy between the true and estimated ages is possibly explained by four mechanisms described below.

First, the discrepancy can be caused by a different physical and chemical property between ^40^Ar_R_ and K in K-rich minerals. Previously, it has been reported that Ar–Ar ages for approximately 120 µm thick muscovite obtained via the crushing method were older than those obtained via the heating and laser ablation methods [54]. This phenomenon has been explained by the preferential release of ^40^Ar_R_ compared to ^39^Ar_K_ during crushing process; ^40^Ar_R_ is loosely retained in minerals compared to ^39^Ar_K_, which is contained in the crystal lattice of K-bearing minerals. However, Ca/K values in the later crushing steps of GU103-1 and GU103-2 higher than 1 are inconsistent with the significant presence of a K-rich mineral.

The second mechanism to explain the discrepancy is recoil of ^39^Ar_K_ in minerals trapped in fluid inclusion via irradiation and release of ^40^Ar_R_ via alteration between fluids and minerals in the fluid inclusion. During neutron irradiation, ^39^Ar_K_ recoils over several hundreds of nanometres. When the K-bearing minerals are very small e.g. less than 1 µm, a significant amount of ^39^Ar_K_ is recoiled into the fluid. Conversely, ^40^Ar_R_ in minerals is slowly released over a few billion years of alteration at temperatures close to room temperature. Younger ages from K-bearing minerals (1027 ± 41 Ma) than expected from the depositional age (approx. 1526 Ma) were reported by a previous study of Ar–Ar dating of middle Proterozoic quartz with K-bearing minerals as the main K reservoir in fluid inclusions using crushing and heating methods (approx. 1526 Ma; [57]). This gap can be explained by ^40^Ar_R_ degassing from minerals to fluid inclusion via alteration being a more significant effect for ^40^Ar_R_/^39^Ar_K_ than the recoil of ^39^Ar_K_. Another previous study reported ^40^Ar-poor and ^39^Ar_K_-rich argon gas extracted from a 3.5 Ga hydrothermal quartz sample during heating experiments [31] that may also be explained by this mechanism. This mechanism makes the apparent age of fluid inclusions containing K-bearing minerals extracted by crushing experiments appear older than the depositional age due to the addition of ^40^Ar. In this case, a calculation of the depositional age from the quartz sample additionally requires the K abundance of the minerals, the ^40^Ar_R_/^39^Ar_K_ value of the mineral, and the contributions of ^39^Ar_K_ via recoil. These additional datasets can be provided by an additional heating analysis of an irradiated sample and a crushing analysis of the non-irradiated sample. However, K-rich minerals are likely to be efficiently trapped in larger inclusions, and the K-rich composition extracted from later crushing steps in this study is likely to be inconsistent with an explanation using this mechanism.

Third, in the case of a K-rich component contributed primarily by K-rich fluid inclusions with minimal contributions from minerals, only the ^39^Ar_K_ recoiling out from the inclusions would possibly make the apparent age older than the depositional age. This K-loss process is insufficient to affect the quartz samples because neutron-irradiated quartz grains have sizes of several hundreds of micrometres.

Fourth, in the case of EM3 contributed by K-rich fluid inclusions, the different ^40^Ar_E_/^38^Ar_Cl_ values between EM2 and EM3 can explain the discrepancy. The three-component mixing model and the regression plane assume a constant ^40^Ar_E_/^38^Ar_Cl_ ratio in each sample. When this assumption is inappropriate, the obtained age is different from the true formation age. The gap between the apparent age of 2.7 Ga and the expected depositional age of 2.4 Ga implies that the assumption of the three-component mixing model is inappropriate including GU103-1 due to the non-constant ^40^Ar_E_/^38^Ar_Cl_ value between the Cl-rich endmember and the K-rich endmember.

We cannot clearly decide which mechanism is the most suitable to explain the discrepancy of the four described above due to the absence of a heating analysis. However, the Ca/K values and the degassing profiles suggest that the last mechanism is the most likely of the four.

#### 5.3.2. Variations of ^40^Ar_E_/^38^Ar_Cl_ in a single sample

**Figure 11.**
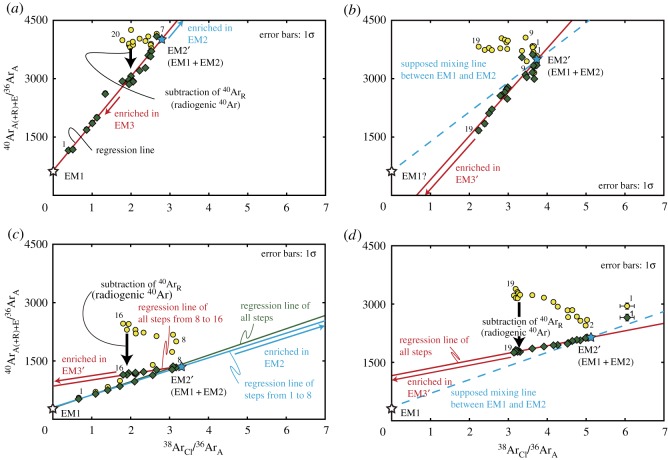
^40^Ar_A(+R)+E_ and ^38^Ar_Cl_ normalized by ^36^Ar_A_ at 2.4 Ga: (*a*) GU84, (*b*) GU91, (*c*) GU103-1 and (*d*) GU103-2. All samples except GU103-1 show a single mixing line, while GU103-1 shows two mixing lines between EM1 (open star) and EM2 indicated by the light blue line, and between EM2′ (light blue star) and EM3′ indicated by red line. In GU103-1, the total regression line (green line) is similar to that of steps 1–8; however, that of steps 8–16 shows a significantly different mixing line, indicating that the K-rich endmember of GU103-1 has a ^40^Ar_E_/^38^Ar_Cl_ value higher than approximately 468. The light blue dashed lines indicate supposed mixing line between EM1 and EM2. The other symbols in this figure correspond to those in figure 7.

Assuming that the analysed hydrothermal quartz and all the fluid inclusions formed at 2.4 Ga, the diagrams of ^40^Ar_A+E_/^36^Ar_A_ and ^38^Ar_Cl_/^36^Ar_A_ for GU103-1 show two mixing lines between EM1 mixed with EM2 (EM2′) and EM3 mixed with EM1 (EM3′) and between EM1 and EM2, suggesting that the ^40^Ar_E_/^38^Ar_Cl_ ratio of EM3 is different from that of EM2 in these samples (figure 11*c*). The bulk regression line for GU103-1 shows small uncertainty and a ^40^Ar_E_/^38^Ar_Cl_ value of 340 ± 37 (95% confidence). However, the mixing line between EM2′ and EM3′ of GU103-1 indicates that the ^40^Ar_E_/^38^Ar_Cl_ value of EM3 is higher than approximately 468, approximately 40% higher than the ^40^Ar_E_/^38^Ar_Cl_ value from the total regression line, based on a slope of a line connecting between EM1 and a data point of the last step. The EM3 of GU103-2 also shows a similar minimum ^40^Ar_E_/^38^Ar_Cl_ value of approximately 464. These data clearly show that the fluid inclusions with apparently constant ^40^Ar_E_/^38^Ar_Cl_ value can involve different ^40^Ar_E_/^38^Ar_Cl_ components. These types of fluid inclusions induce misinterpretation when constraining the mineral formation age and the ^40^Ar_A_/^36^Ar_A_ value of the palaeoatmosphere. However, apparent ages of 2114 ± 312 Ma from GU84 and 2701 ± 43 Ma from GU103-1 obtained by the three-component mixing model varies around the expected depositional age of 2.4 Ga. These variations may imply that all the primary fluid inclusions formed at approximately 2.4 Ga and that the ^40^Ar_E_/^38^Ar_Cl_ value of the mixing line between EM2′ and EM3′ is slightly different from that of EM2.

### 5.4. Origins of the fluid components

The continuous change of the extracted argon gas composition from the quartz corresponds to the sizes and generation of fluid inclusions, as discussed in §5.2.5, representing the evolution of Ongeluk hydrothermal fluid. The assumption for the quartz depositional age of 2.4 Ga enables the following interpretation. EM3 which is trapped in smaller inclusions of early generations is enriched in K with various Ca/K ratio among samples and has higher or similar ^40^Ar_E_/^38^Ar_Cl_ values compared with EM2. The ^40^Ar_E_/^36^Ar_A_ value of EM3 is lower than that of EM2 (when the ^40^Ar_A+E_/^36^Ar_A_ value is similar to EM2, obtained age of approximately 0 Ma for GU84 and approximately 1.9 Ga for GU103-1). EM2, which is trapped in moderate-sized inclusions of moderate generations has a Cl- and ^40^Ar_E_-rich composition and is poor in K. However, EM2 of all samples except GU84 shows a lower Ca/K value than 1, indicating a higher abundance of K than of Ca. The lower abundances of the two major cations in the fluid imply that EM2 has an Na-rich composition, i.e. EM2 is an Na-rich fluid. The clear presence of ^40^Ar_E_ in EM2 and EM3 indicates a lack of seawater and surface water in the Ongeluk hydrothermal system. A hydrothermal system separated from ambient seawater implies a presence of a cap rock, such as the sulfate minerals in the modern seafloor hydrothermal system (e.g. [64]). The hydrothermal quartz itself possibly effected as a cap rock. The highest ^40^Ar_A+E_/^36^Ar_A_ value of all the samples is 4100 for GU84 indicating that the ^40^Ar_E_-rich component could be derived from a fluid that reacted with the 100–200 Ma older sedimentary rocks located stratigraphically below the Ongeluk Formation and/or a fluid containing volatiles degassed from the plume source of the Ongeluk volcanism. The former candidate is supported by the presence of ^13^C-depleted calcite (–31.9‰) in the Ongeluk interpillow jaspilites, indicative of the decomposition of organic matter in the underlying sedimentary rocks during seafloor hydrothermal circulation [36]. The latter candidate would also be supported by the absence of ^40^Ar_A+E_/^36^Ar_A_ values much over 4000 in the samples when the ^40^Ar_A+E_/^36^Ar_A_ value of the Ongeluk plume source is assumed to have been similar to those of 2.4 Ga oceanic island basalts with values of approximately 4000 [65]. In previous studies of fluid inclusions in hydrothermal quartz with MORB-like basalt in the 3.5 Ga Dresser Formation, West Australia, the highest ^40^Ar_A+E_/^36^Ar_A_ value was consistent with the expected ^40^Ar/^36^Ar value of MORB source mantle at 3.5 Ga [14,31]. Therefore, obtaining the highest ^40^Ar_A+E_/^36^Ar_A_ of 4100 similar to the expected value for the Ongeluk plume source supports the idea that the fluid inclusions formed due to hydrothermal circulation driven by the Ongeluk volcanism. EM1 shows the atmospheric composition compared with EM2 and EM3. In particular, EM1 of GU103-1 shows a ^40^Ar_A+E_/^36^Ar_A_ value similar to the atmospheric value and fluid inclusions trapped in hydrothermal/relict minerals in meta-gabbros of modern oceanic crusts [66]. The atmospheric component probably corresponds to the 2.4 Ga Ongeluk seawater because the seafloor hydrothermal alteration is well preserved in the Ongeluk lavas [33,35] and the compositional variation of the fluid inclusions in the hydrothermal quartz points to the presence of a seawater-like endmember [12,13]. However, the obtained ^40^Ar_A+E_/^36^Ar_A_ values with a significant difference between that from GU84 and GU103-1 are higher than modern atmospheric value and similar to that of the fluid inclusions in meta-gabbros, suggesting the small amounts of seawater in the Ongeluk hydrothermal system.

**Figure 12.**
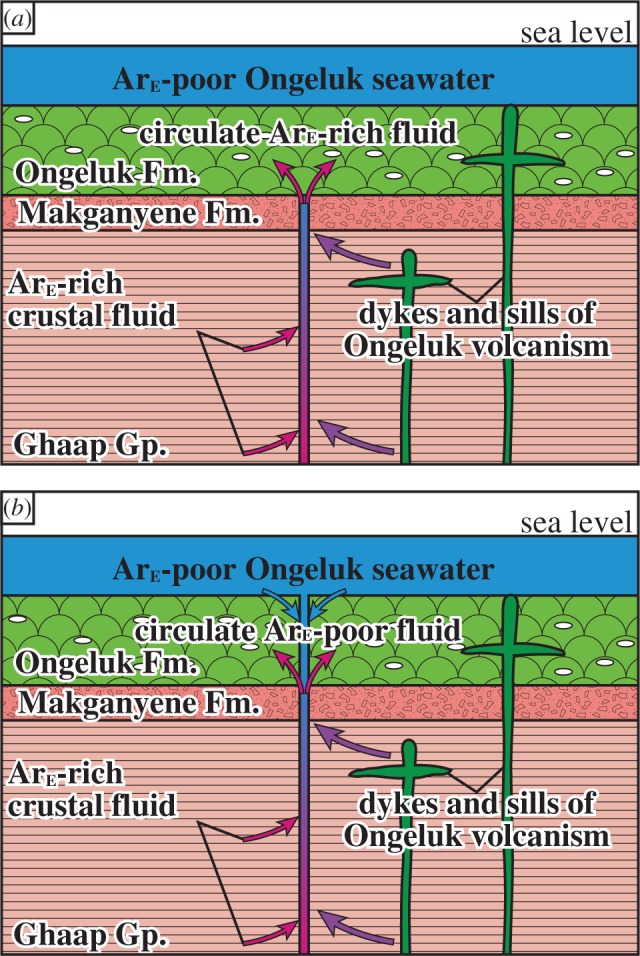
Schematic images of the hydrothermal systems hosted by the 2.4 Ga Ongeluk volcanism. (*a*) The rocks in the hydrothermal system consist of the Ongeluk lavas (900 m thick), the Makganyene diamictites (7–500 m thick) and the sedimentary rocks of the Ghaap Group (approximately 2500 m thick). In the early hydrothermal system, circulated fluid mainly is composed of crustal fluid from sedimentary rocks and magmatic fluid from Ongeluk volcanism. The mixed fluid took place extensively in the Ongeluk Formation, which would have precipitated the hydrothermal quartz within the cavities and interstitial open spaces in the lavas. (*b*) In the later hydrothermal system, the downwelling Ongeluk seawater mixed with the crustal fluid and/or with magmatic fluids.

Previously, the seafloor hydrothermal circulation hosted by the Ongeluk volcanism was primarily explained by interactions between seawater-derived hydrothermal fluids and the volcanic rocks (e.g. [12,13,33]). However, the argon isotopes of the hydrothermal quartz in this study could provide new constraints on the fluid sources during the seafloor hydrothermal circulation; there was fluid mixing between the deep crustal fluid derived from the underlying sedimentary rocks and the plume source of the Ongeluk volcanism without the involvement of the Ongeluk seawater. After the hydrothermal alteration without seawater, the (a little) Ongeluk seawater is mixed with crustal fluid possibly along the faults (figure 12).

## 6. Conclusion

Stepwise crushing extraction of the quartz samples reveals that the fluid inclusions with different size are different argon compositions, ^36^Ar-rich larger inclusions, ^38^Ar_Cl_- and ^40^Ar-rich moderate-sized ones and ^39^Ar_K_- and ^40^Ar-rich smaller fluid inclusions.

The 3D regressions for the obtained argon isotope data clearly indicate that the compositional variations are formed by three major components: K-rich fluid, excess argon-rich fluid and atmospheric argon-rich fluid.

An age of 2732 ± 45 Ma for GU103-1 was obtained from the 3D plot of the three-component mixing model, which is significantly older than reported ages for the Ongeluk volcanism (2425.5 ± 2.6 Ma). The apparent gap of approximately 300 Ma was probably caused by the different ^40^Ar_E_/^38^Ar_Cl_ value of K-rich fluid from the Cl-rich fluid.

It is difficult to constrain a depositional age based on Ar–Ar dating by crushing method using only fluid inclusion containing excess argon due to the large variation of ^40^Ar_E_/^38^Ar_Cl_.

The highest ^40^Ar_A+E_/^36^Ar_A_ value (4100) of the age-corrected data as 2.4 Ga from the Ongeluk fluid inclusions probably represents the contribution of the deep crustal fluid from older sediments below the Ongeluk Formation and derived from the plume source of the Ongeluk volcanism, suggesting that these fluid inclusions were formed at 2.4 Ga.

The presence of excess argon indicates (a) little seawater contribution in the Ongeluk hydrothermal system. The Ongeluk hydrothermal fluid evolves from crustal fluid and magmatic fluid without seawater into a little seawater and these fluids.

## Supplementary Material

Argon isotopes of quartz samples by crushing.

## Data accessibility

Raw data of the argon isotopes have been uploaded as the electronic supplementary material.
